# Management of undifferentiated adrenal gland metastases from malignant melanoma: case report

**DOI:** 10.3389/fonc.2024.1419827

**Published:** 2024-08-20

**Authors:** Hannah Shortreed, Nishigandha Burute, Olexiy Aseyev

**Affiliations:** ^1^ Department of Undergraduate Medical Education, Northern Ontario School of Medicine University, Thunder Bay, ON, Canada; ^2^ Department of Diagnostic Imaging, Thunder Bay Regional Health Sciences Centre, Thunder Bay, ON, Canada; ^3^ Department of Medical Oncology, Cancer Care Northwest, Thunder Bay, ON, Canada; ^4^ Department of Medical Oncology, Thunder Bay Regional Health Sciences Centre, Thunder Bay, ON, Canada

**Keywords:** malignant melanoma, adrenal gland metastasis (AGM), immunotherapy, metastasectomy, case report

## Abstract

Adrenal gland metastases from malignant melanoma are a common but poorly characterised condition. Their lack of consistent clinical features and poor response to immune checkpoint inhibitors pose a significant diagnostic and therapeutic challenge to practitioners. This case report describes a 78-year-old male with no prior history of melanoma presenting with nonspecific abdominal symptoms and unintentional weight loss who was found to have undifferentiated bilateral adrenal gland metastases from malignant melanoma. Despite ongoing investigations, the primary site of the adrenal gland metastases remained unknown, prompting the consideration of primary adrenal melanoma as a diagnosis. The patient underwent four cycles of treatment with immune checkpoint inhibitors, nivolumab and ipilimumab, followed by maintenance therapy and subsequent adrenal metastasectomy. Despite therapeutic efforts, the patient’s tumour was resistant to treatment and became undifferentiated. The patient continued with palliative care until his death, more than three years after the onset of symptoms. The clinical features, pathophysiology, diagnosis, treatment, and prognosis of this patient’s disease are discussed in detail to help inform the management of similar cases.

## 1 Introduction

Melanomas are cancers originating in melanocytes and can occur in various tissues, such as the epidermis, dermis, mucosa, and uvea (1, 2). Many melanomas arise from preexisting nevi at the dermal-epidermal junction and display characteristic signs of malignant transformation, including asymmetry, irregular borders, recent enlargement, colour variations, surface changes (i.e. bleeding), thickening, development of satellite pigmentation, and inflammation (2). Early metastases typically spread via the lymphatic system, resulting in regional lymphadenopathy, before invading the bloodstream and causing more disseminated disease (3). This metastatic spread to visceral sites (i.e. lung) is correlated with poor outcomes, however, in recent years, the use of targeted therapeutic agents, such as immune checkpoint inhibitors (ICIs), has improved the prognosis of metastatic melanoma considerably (4–7). This includes the use of programmed death-1 (PD-1) pathway inhibitors, such as nivolumab and pembrolizumab, and cytotoxic T-lymphocyte-associated protein 4 (CTLA-4) inhibitors, such as ipilimumab (5–7).

Despite advances in the immunotherapy-based treatment of metastatic melanoma, certain metastatic sites, including the adrenal glands, are highly resistant to ICIs (8). Notably, the adrenal gland is the fourth most common site for metastatic melanoma, behind the lung, liver, and bone, but due to the absence of obvious biochemical changes and clinical symptoms, involvement of the adrenal gland is often only an incidental finding during imaging or post-mortem examination (8). Overall, it is hard to appreciate the true incidence of adrenal gland metastasis (AGM) from malignant melanoma, and the progression of patients with this disease is poorly reported in the literature (9). This case report describes a 78-year-old male presenting with abdominal pain, exertional dyspnea, nausea, and unintentional weight loss, who was found to have bilateral adrenal gland masses on imaging and later confirmed to be undifferentiated malignant melanoma with an unknown primary site. The unique clinical presentation of this patient’s disease is explored in detail to help gain a greater understanding of AGM from metastatic melanoma and improve diagnosis and treatment of this condition.

## 2 Case description

A 78-year-old Caucasian male with no prior history of melanoma presented to the Thunder Bay Regional Health Sciences Center (TBRHSC) emergency room (ER) in November 2020 with left upper quadrant pain, recent unintentional weight loss of 25-30 lbs, nausea, and exertional dyspnea. He endorsed belching, abdominal bloating, flatus, poor appetite, and irregular bowel movements with occasional diarrhea and constipation. He denied any additional symptoms.

The patient was a non-smoker and drank alcohol infrequently. His medical history was significant for a one-month hospitalization in his 30s following a propane explosion. This resulted in extensive burns to his arms, face and chest requiring skin grafting. In 2017, he had a mole excised from an unknown location, citing cosmetic concerns. No pathology report is available from that procedure, and it is not known whether it was a new growth undergoing dysplastic changes or if it was a long-standing mole. He endorsed no significant history of sun nor artificial ultraviolent light exposure. His family history was significant for his mother having died of colon cancer in her mid-80s and two of his brothers being diagnosed with cancer in their 70s; one with pancreatic cancer treated by Whipple’s resection and the other with lymphoma.

The patient’s left upper quadrant pain was previously investigated in August 2020 via endoscopy. A patulous gastroesophageal junction was observed, and biopsy of this site returned negative for any pathology. Gastroesophageal reflux disease (GERD) was suspected, and he began pantoprazole 40 mg PO daily. This was ineffective in managing his symptoms, prompting his visit to the ER in September wherein he was found to be hypertensive (blood pressure 142/96 mmHg) with abdominal distention and bloating that prevented deep palpation and detection of organomegaly. All other physical findings and vitals were within normal limits.

## 3 Clinical approach and timeline

**Figure 1:**
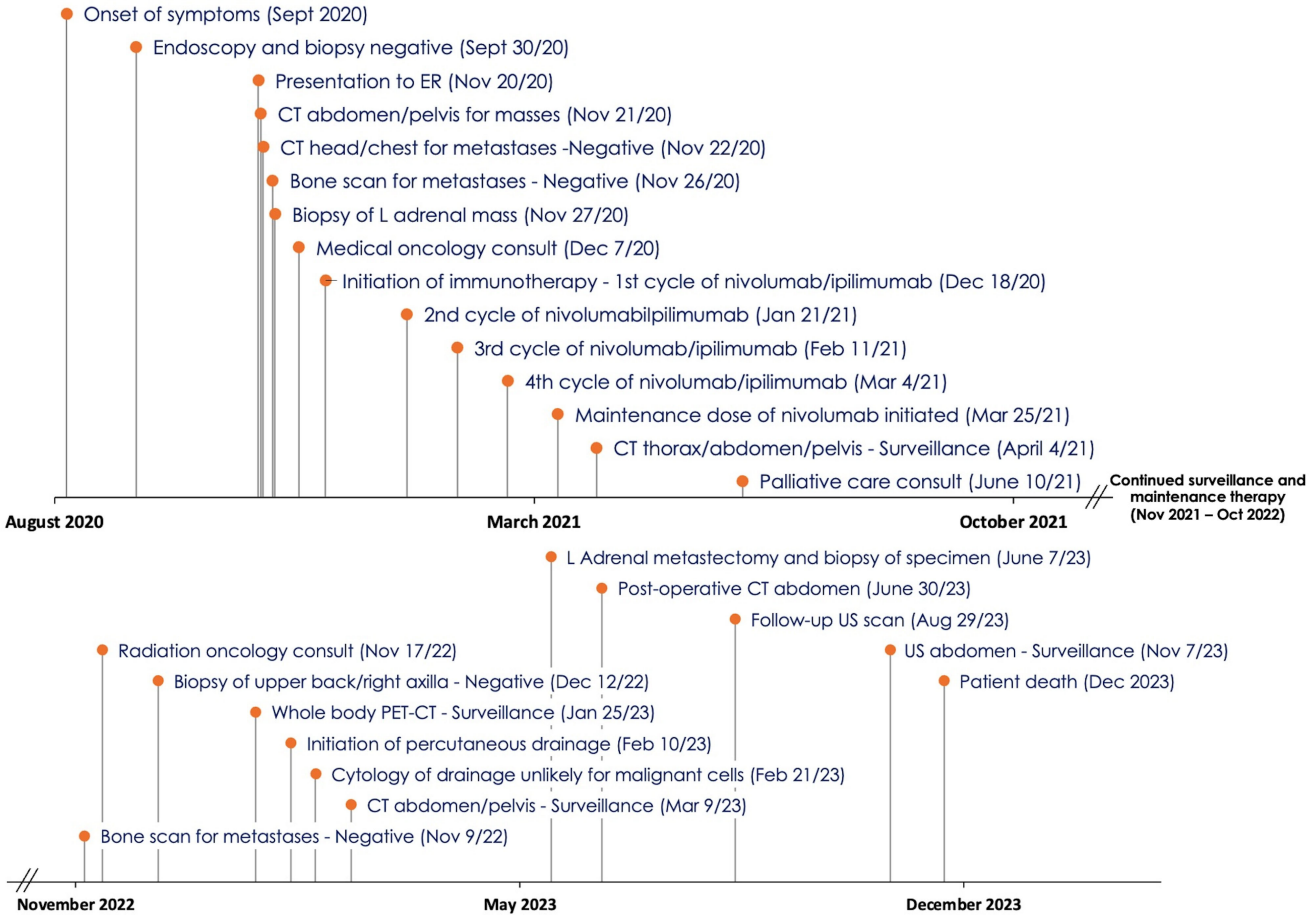
Timeline with data from course of care.

Please refer to **Figure 1** for a timeline with data from the related episodes of care.

### 3.1 Diagnostic assessment

Bloodwork revealed decreased hemoglobin (128 g/L) and hematocrit (38%) and elevated sodium (156 mEg/L) and bicarbonate (32 mEq/L). Venous blood gases revealed low bicarbonate (11 mEq/L) and elevated total CO_2_ (35 mEq/L). Urinalysis showed protein at 0.15 g/L and occult blood at 0.3 mg/L. All other laboratory findings were within normal limits.

**Figure 2:**
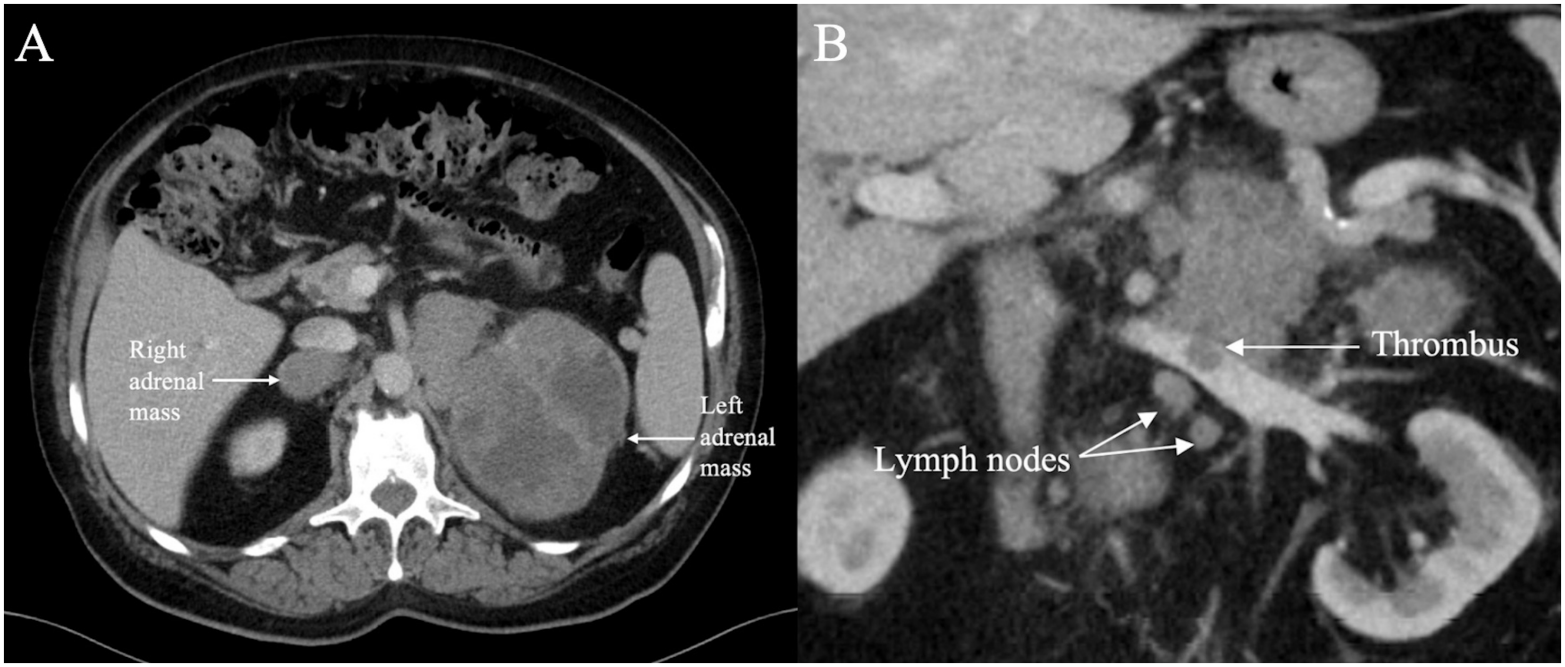
Axial contrast enhanced CT **(A)** through the upper abdomen in November 2020 shows a 11 x 9.9 cm lobulated heterogeneously enhancing solid cystic mass in the left adrenal gland and a 4.6 x 3.4 cm similar appearing mass in the right adrenal gland (indicated by arrows). Coronal contrast enhanced CT **(B)** through the upper abdomen in November 2020 shows an intraluminal filling defect in the left renal vein consistent with thrombus associated with the left adrenal mass. A few suspicious lymph nodes are present in the vicinity.

An initial computed tomography (CT) scan of the abdomen and pelvis revealed heterogeneously enhancing solid mass lesions measuring 4.6 x 3.4 cm in the right adrenal gland and 11 x 9.9 cm in the left adrenal gland displacing the spleen and left kidney (**Figure 2**). Additionally, a small non-occlusive tumour thrombus was observed in the left renal vein. Adrenal adenocarcinoma was suspected at this time.

No metastases were found on a subsequent head and chest CT and whole-body bone scan. An ultrasound (US) guided biopsy of the left adrenal mass revealed a poorly differentiated malignant spindle cell tumour positive for vimentin, Melan-A, S-100 and HMB-45. The mass was negative for additional immunohistochemical markers. Due to insufficient DNA, molecular testing for BRAF gene mutation was not possible. The pathology report supported the diagnosis of an undifferentiated malignant neoplasm, most likely a melanoma, that had metastasized to the adrenal glands from an unknown primary site. Consultation with medical oncology supported this diagnosis, and the patient began an immunotherapy regimen in December 2020. Investigations for a primary site, including a skin biopsy of the upper back and right axilla in December 2022, returned negative, and a diagnosis of primary adrenal melanoma was considered. Throughout this patient’s disease, the tumours remained confined to the adrenal glands.

### 3.2 Therapeutic intervention

The patient underwent four cycles of nivolumab 90 mg IV and ipilimumab 250 mg IV. Following the first cycle, he developed a rash and was administered diphenhydramine 50 mg PO. The rash persisted, and he was started on prednisone 90 mg PO daily with a taper of 10 mg every subsequent week with successful resolution of the rash. From March 2021 – October 2021, the patient remained on a maintenance dose of nivolumab 240 mg IV administered on days one and 15 of a four-week cycle. In November 2021, this was changed to 480 mg IV once per four-week cycle due to discomfort at his peripherally inserted central catheter (PICC). By his last cycle of treatment in September 2023, he had received a total of 22 cycles of maintenance nivolumab. Overall, he tolerated immunotherapy well with minimal side effects.

**Figure 3:**
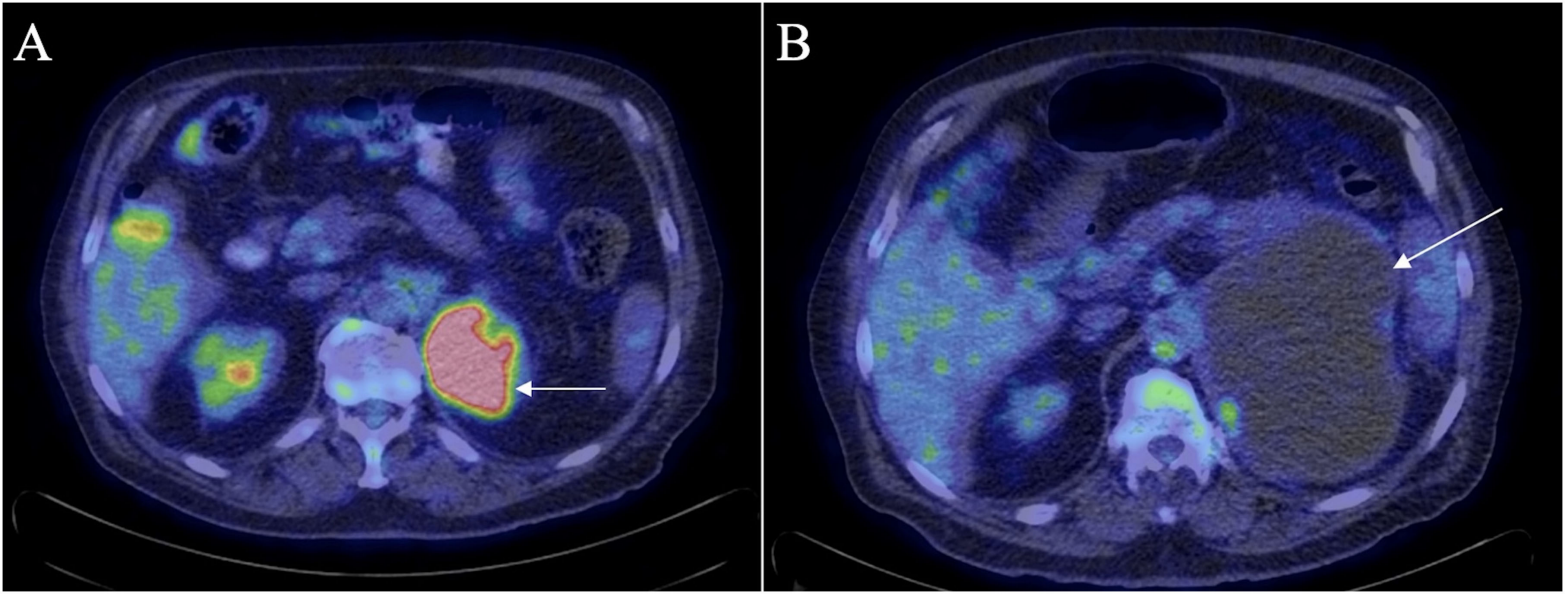
PET-CT scan in June 2021 **(A)** shows residual uptake within the periphery of the left adrenal mass (arrow) with an SUV of 14.7. There was no significant metabolic activity in the right adrenal mass. PET-CT scan in January 2023 **(B)** shows enlargement of the left adrenal mass (arrow) which appears almost completely cystic with reduced volume and extent of metabolic uptake within the mass as compared to the previous PET-CT scan.

In January 2023 a whole-body positron emission tomography (PET)-CT scan was compared to a previous PET-CT scan from June 2021 (**Figure 3**). This scan showed significant interval enlargement of the left adrenal metastasis, now measuring 15 x 10 cm, and indicated that it was predominantly cystic. Focal areas of increased uptake were seen along the periphery of the lesion [standardized uptake value (SUV) of 6.3]. The overall volume of residual hypermetabolic disease was reduced, and no other evidence of hypermetabolic disease was observed, including in the right adrenal mass.

In November 2022, palliative radiation therapy was considered but advised against due to the large cystic volume of the tumour. Instead, percutaneous drainage was recommended to help reduce the size of the cyst. Subsequent cytology of the drainage revealed degenerative and atypical cells. No cancerous cells were noted; however, the sample was unsatisfactory for a complete evaluation owing to blood and debris.

**Figure 4:**
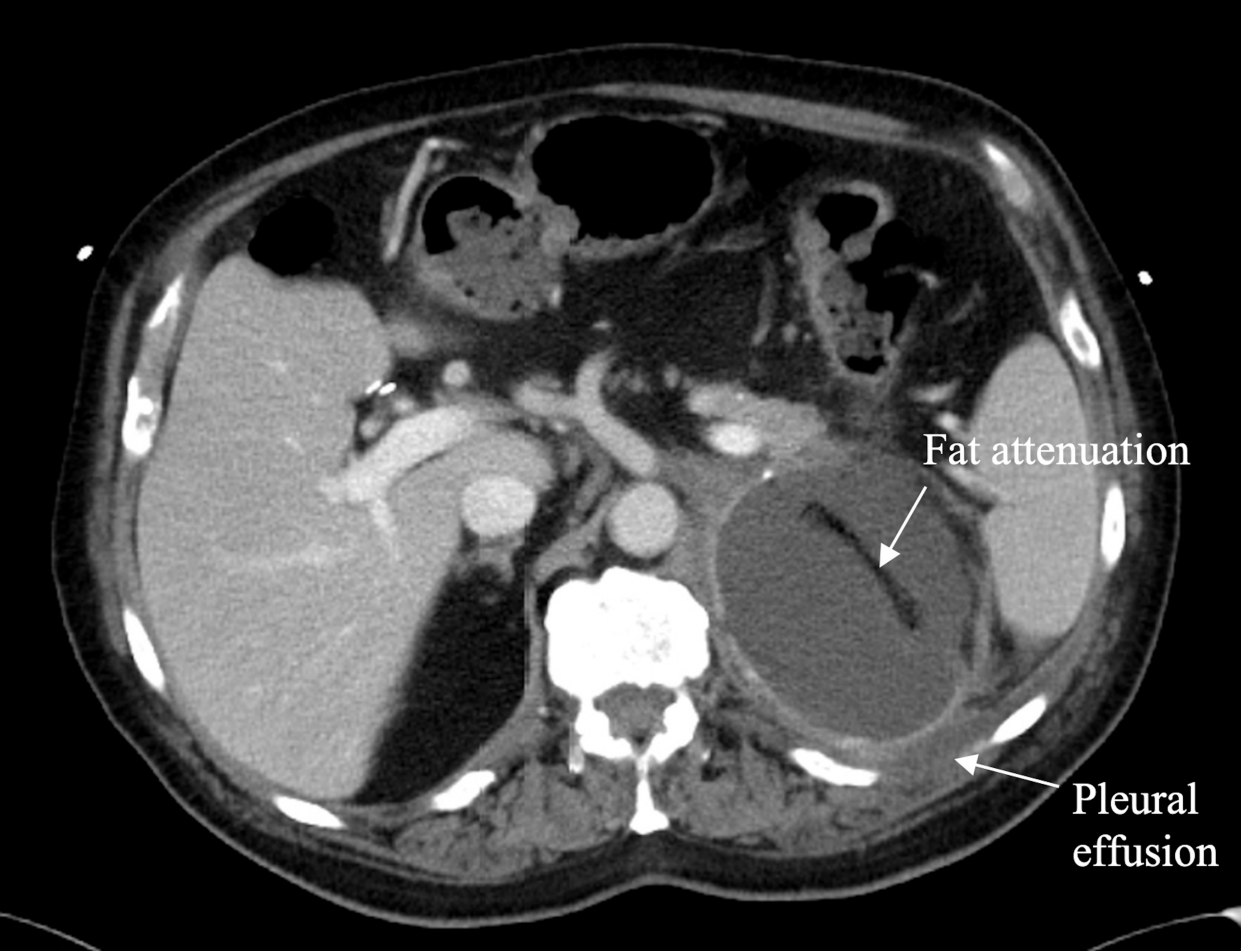
Axial contrast enhanced CT through the upper abdomen in June 2023 shows complete replacement of the left adrenal gland by a well circumscribed fluid attenuation mass with smooth margins measuring 9.9 x 7.9 x 7.8 cm. A linear area of fat attenuation is seen within the mass along the path of a previously placed pigtail catheter. There is a small left pleural effusion. The right adrenal mass has resolved, and the right adrenal gland appears normal.

Since the patient had been stable with no evidence of further metastatic disease, the decision was made to perform an open left adrenal metastasectomy. In June 2023, the left adrenal mass and surrounding peri-tumour tissue were removed without complications. A specimen taken from the adrenal mass was found to have abundant areas of necrosis and hemorrhage as well as a fibrotic periphery with chronic inflammatory cell infiltrate. No normal adrenal tissue could be identified. Immunohistochemical examination showed that tumour cells were positive for vimentin, synaptophysin, CD56, and CD10. Of note, they were now nonreactive for Melan-A, HMB-45, and S100. The left peri-renal and renal margins were positive, with poorly differentiated high-grade malignant spindle cell tumours identified in each. A postoperative CT scan performed showed an ongoing left adrenal fluid attenuation mass measuring 9.9 x 7.9 x 7.8 cm, and a normal-appearing right adrenal gland (**Figure 4**). As the smaller of the two, the right adrenal mass was only observed and never biopsied. It did not undergo specific management but showed a resolution in response to immunotherapy at the time of the metastasectomy.

### 3.3 Follow-up and outcomes

In September 2023 the patient appeared alert and orientated with no signs of distress. He remained active, although he noted an increase in fatigue, weakness, and lack of mobility. He reported absent to mild anxiety, depression, appetite fluctuation, constipation, and sleep disturbances [Edmonton symptom assessment system (ESAS) score of 0-2]. Additionally, he was experiencing ongoing pain near the surgical site (managed by naproxen 500 mg PO twice per day) and minimal, but continuous blood loss from the surgical incision due to incomplete wound healing. In November 2023, an US scan of the abdomen revealed that the left suprarenal mass now measured 16.8 x 11.5 x 10.2 cm.

Due to the progressive enlargement of his tumour despite both surgical and immunotherapeutic interventions, and the contraindications towards initiating radiation therapy, the patient could not be recommended any further curative treatment. He was admitted to a palliative unit and received hospice care until his death in early December 2023. While no autopsy was completed, it is suspected that his death resulted from a combination of ongoing blood loss, compression of adjacent structures from tumour progression, and sepsis.

## 4 Discussion

AGM from malignant melanoma are common, however, due to the variability or absence of symptoms in affected individuals, many cases are only discovered incidentally during imaging or post-mortem autopsy (8). One of the key strengths of this report lies in the multi-disciplinary approach to managing this common yet somewhat obscure diagnosis in a patient who presented with nonspecific symptoms. This approach enabled the patient to receive a comprehensive diagnostic assessment, evaluation of treatment modalities, and ongoing care despite the therapeutic difficulties associated with this diagnosis. One of the key limitations of this approach was the uncertainty around the identification of a primary site and the absence of a pathology report from the mole excised roughly three years before the onset of symptoms. As a result, this patient’s diagnosis of malignant melanoma cannot be confirmed, and the possibility of this being a case of primary adrenal melanoma needs to be considered.

The lack of knowledge surrounding the pathophysiology, diagnosis, and treatment of AGM from malignant melanoma pose a significant challenge to practitioners (8). Regarding pathophysiology, one hypothesis suggests that the adrenal gland, specifically the adrenal cortex, is a common site for metastases due to its rich concentration of glucocorticoids (10). Since human melanoma cells express a large number of glucocorticoid receptors, it is thought that they metastasize to this site due to their high affinity for the glucocorticoid rich adrenal cortex (10). Moreover, once the adrenal gland has been infiltrated, corticosteroids produced there may play a key role in helping the tumour cells evade immune response; glucocorticoids have a well-described immunosuppressive effect wherein they impede lymphocyte proliferation and function, limit the activity of major histocompatibility complex (MHC) class I and II, decrease the ability of T cells to recognize tumour cells, and even upregulate the protein β-catenin which contributes to immune cell exclusion in the tumour’s microenvironment (8). Additionally, androgens and catecholamines produced in the adrenal gland can block and diminish immune cell proliferation, cytotoxic capacity, and cytokine production (8). Combined, the chemoattractant and immunosuppressive properties of the adrenal gland may make it an ideal site for metastatic melanoma (8, 10).

Regarding the diagnosis of AGM, clinical presentation can vary widely, with some patients reporting weight loss, nausea, constipation, loss of appetite, generalised weakness, dyspnea, and even generalised pruritus (coupled with abnormal liver function tests) that prompt abdominal CT imaging (11, 12). Moreover, adrenal masses may be found incidentally in patients with unrelated symptoms; a 40-year-old woman with a history of subungual malignant melanoma who presented with recurrent urinary tract infections was referred for a US scan of her kidneys that revealed an adrenal gland mass (12). This mass was later identified as metastatic melanoma (12). In our case, the patient had no history of malignant melanoma and presented with weight loss and nonspecific abdominal symptoms that were initially attributed to GERD before imaging confirmed the presence of bilateral adrenal masses. These descriptions highlight the variability in the clinical presentation of AGM and help explain why it is often only an incidental finding (8).

In addition to clinical presentation, diagnostic features of AGM on CT include a diameter greater than 5 cm, central or irregular sites of haemorrhage and/or necrosis without a lipomatous component, and bilateral gland involvement (12). The patient described in this case report had all these characteristics, except for the mass on the right adrenal gland, which measured 4.6 cm in diameter. Due to the absence of a primary site and no prior history of melanoma, it was proposed that this could be a case of primary adrenal melanoma. This condition is rare and has several criteria that must be met for diagnosis: unilateral adrenal gland involvement; a lack of melanomas elsewhere in the body; no previous surgical resection of pigmented skin, eye, or mucosal lesions; and the absence of obscure pigmented lesions, best confirmed by autopsy (13). Due to bilateral involvement of the adrenal glands and prior mole excision in 2017, the case described here does not meet the criteria (13). Therefore, metastatic disease is more likely.

Immunohistochemical markers and melanoma driver mutations, such as the proteins Melan-A, HMB-45, and S100, as well as the BRAF gene, respectively, provide further diagnostic information for patients with suspected malignant melanoma (14–17). Notably, the patient in this case report was originally positive for Melan-A, HMB-45, and S100 in the left adrenal gland; however, when his tumour was excised approximately 2.5 years later, it was found no longer reactive for these markers. The significance of this change is that it indicates dedifferentiation and subsequent undifferentiation of the AGM despite treatment efforts (14). Historically, the prognosis of patients with metastatic melanoma to the adrenal gland is poor, and data from 15 years ago indicate that median overall survival can be as short as six months (9). The one-year survival rate for all sites of metastatic melanoma was also low, with only 15-25% of the patients surviving (18). With the advent and implementation of ICIs, such as nivolumab and ipilimumab, as well as targeted therapy agents, the five-year survival of metastatic melanoma has increased to over 50% in recent years (18, 19). Moreover, research suggests that patients with dedifferentiated and undifferentiated melanoma can have a favourable response to and benefit from ICI therapy (20).

Despite treatment efforts, the patient’s tumour described in this case report became undifferentiated and had grown significantly since his initial diagnosis. One potential reason for these changes is the site of the metastases itself. Studies have shown that AGMs have a significantly decreased disease control rate to ICIs (calculated from the complete response, partial response, and stable disease values reported for ICI therapy) compared to patients without AGM (29% versus 76%, respectively) (8). The same mechanism involved in the pathophysiology of AGM may explain this resistance; the naturally immunosuppressive environment of the adrenal glands limits the ability of the patient’s immune system to mount a response against tumour cells (8). Resultantly, tumour cells can evade immune surveillance and proliferate, even with the implementation of ICIs (8).

Due to immunotherapeutic resistance of the adrenal gland, adrenalectomy is an alternative option that has the potential to prolong survival in patients with isolated AGM (21). However, many clinicians remain unconvinced of the benefit of nonpalliative surgical intervention for metastatic disease, making this approach somewhat controversial (22). Ultimately, the decision to proceed with surgical intervention must be considered by multidisciplinary teams on a case-by-case basis to ensure the best patient outcomes, as was done for the case described in this report. While the procedure was without complications, the postoperative CT scan showed an ongoing left adrenal mass that could have represented further metastatic spread (**Figure 4**). However, it was not possible to examine the lesion further as a new biopsy was not taken prior to the patient’s death.

In closing, this case report describes a 78-year-old male who presented with nonspecific abdominal symptoms and weight loss who was later confirmed to have bilateral AGM from malignant melanoma despite no prior history of melanoma. He was managed with ICIs and underwent a left adrenal metastasectomy, however his tumour was resistant to treatment and became undifferentiated prior his death which occurred more than three years after the onset of symptoms. Ultimately, this patient’s case highlights the challenge of managing AGMs from malignant melanoma and reinforces the need for further advancement in the diagnosis and treatment of this condition to improve patient prognosis.

## Data Availability

The original contributions presented in the study are included in the article/supplementary material. Further inquiries can be directed to the corresponding author.
